# Parental genetic distance and patterns in nonrandom mating and seed yield in predominately selfing *Arabidopsis thaliana*

**DOI:** 10.1007/s00497-013-0228-5

**Published:** 2013-07-11

**Authors:** Ann L. Carlson, Hui Gong, Christopher Toomajian, Robert J. Swanson

**Affiliations:** 1 Department of Biology, Valparaiso University, Valparaiso, IN 46383 USA; 2 Department of Mathematics and Computer Science, Valparaiso University, Valparaiso, IN 46383 USA; 3 Department of Plant Pathology, Kansas State University, Manhattan, KS 66506 USA

**Keywords:** Mate choice, Nonrandom mating, Seed yield, Inbreeding, Diallel, Pollen competition

## Abstract

In this study, we ask two questions: (1) Is reproductive success independent of parental genetic distance in predominately selfing plants? (2) In the absence of early inbreeding depression, is there substantial maternal and/or paternal variation in reproductive success in natural populations? Seed yield in single pollinations and proportion of seeds sired in mixed pollinations were studied in genetically defined accessions of the predominately selfing plant *Arabidopsis thaliana* by conducting two diallel crosses. The first diallel was a standard, single pollination design that we used to examine variance in seed yield. The second diallel was a mixed pollination design that utilized a standard pollen competitor to examine variance in proportion of seeds sired. We found no correlation between reproductive success and parental genetic distance, and self-pollen does not systematically differ in reproductive success compared to outcross pollen, suggesting that Arabidopsis populations do not experience embryo lethality due to early-acting inbreeding or outbreeding depression. We used these data to partition the contributions to total phenotypic variation from six sources, including maternal contributions, paternal contributions and parental interactions. For seed yield in single pollinations, maternal effects accounted for the most significant source of variance (16.6 %). For proportion of seeds sired in mixed pollinations, the most significant source of variance was paternal effects (17.9 %). Thus, we show that population-level genetic similarities, including selfing, do not correlate with reproductive success, yet there is still significant paternal variance under competition. This suggests two things. First, since these differences are unlikely due to early-acting inbreeding depression or differential pollen viability, this implicates natural variation in pollen germination and tube growth dynamics. Second, this strongly supports a model of fixation of pollen performance genes in populations, offering a focus for future genetic studies in differential reproductive success.

## Introduction

The events that occur after pollination in angiosperms include pollen germination, pollen tube growth, double-fertilization and the development of the resulting embryo and endosperm. Understanding natural variation in this process is complicated by the sheer number of genetic entities involved. For example, differences in pollen germination and pollen tube growth may be traced back to the male gametophyte genome, but may also be influenced by the condition or the genetics of the paternal sporophyte that preloads pollen grains with nutrients used for the initial stages of pollen tube growth (Stephenson et al. 2003). The female gametophyte also plays roles in guiding pollen tubes during the last stages of reproduction (e.g. (Higashiyama et al. 2001; Higashiyama 2002; Okuda et al. 2009)). All of this takes place in the context of female sporophytic tissue, the pistil, whose condition and/or genetics may interact differently with different pollen populations or seed genotypes.

Understanding natural variation in reproduction is further complicated because individuals may display different and varied phenotypes resulting from heterozygosity and/or inbreeding depression. Plants that are highly heterozygous can produce pollen populations that display very different phenotypes because of segregating alleles. This variation within pollen populations complicates genetic analysis of paternal roles in reproductive success. And in plants that carry genetic load, reproductive success may be context dependent, where selfing or mating with a genetically similar plant can lead to reduced reproductive success. This is likely the result of deleterious recessive alleles pairing and negatively affecting embryo viability, and/or the result of mate discrimination that may have evolved in response to inbreeding depression (Waser et al. 1995; Armbruster and Rogers 2004). For example, in outcrossing plants that have the capacity to self, self-pollinations often (but not always) yield less seed than outcross pollinations (Weller and Ornduff 1977; Cruzan 1989; Aizen et al. 1990; Snow and Spira 1993; Bell et al. 2010) but see (Snow and Spira 1991b, 1993; Sork and Schemske 1992; Jones 1994). Similarly, in mixed pollinations in self-compatible plants, self-pollen often sires a disproportionately low number of seeds, sometimes termed cryptic self-incompatibility (Bateman 1956; Weller and Ornduff 1977; Bowman 1987; Eckert and Barrett 1994; Jones 1994; Hauser and Siegismund 2000, Teixeira et al. 2009) but see (Sork and Schemske 1992; Johnston 1993).

Thus, in outcrossing populations, genetically based differences in reproductive success, parental relatedness and segregating allelic variation are all interlinked, and all may influence reproductive outcomes. Two of these factors can be potentially eliminated by studying plant populations that have historically selfed. As outcrossing populations become increasingly self-fertilizing, they both lose heterozygosity, and their genetic load is purged (Lande and Schemske 1985; Schemske and Lande 1985; Charlesworth and Charlesworth 1987; Lande et al. 1994; Byers and Waller 1999; Crnokrak and Barrett 2002). Thus, historically selfing populations of plants produce more genetically uniform populations of pollen. Selfing populations also become less and less likely to experience strong inbreeding depression, resulting in self-embryo viability eventually approaching (or potentially exceeding) outcross embryo viability. Thus, this system may offer the best opportunity to explore the question of whether natural variation in maternal and paternal function in reproduction develops or persists in the absence of inbreeding depression.

A variety of experimental methods have confirmed separable paternal and maternal roles in differential reproductive success. For example, seed paternity tests in *Raphanus sativus* demonstrate that that some pollen sire more seeds than others across a range of maternal plants (Marshall and Ellstrand 1986, 1988; Marshall 1998; Mitchell and Marshall 1998). Other more direct measures of paternal function, such as pollen tube growth rates, verify variation in male function and its role in differential reproductive success (Snow and Spira 1991a, b; Pasonen et al. 1999; Skogsmyr and Lankinen 1999; Stephenson et al. 2001; Lankinen and Skogsmyr 2002, Lankinen et al. 2009). Maternal roles in differential reproductive success have also been demonstrated using different experimental strategies. For example, diallel analysis verifies significant maternal variation in seed yield in *Ipomopsis aggregate, Companula rapunculoides, Asclepias incarnate* and *Leavenworthia crassa* (Waser et al. 1995; Lyons 1996; Lipow and Wyatt 1999; Good-Avila et al. 2003). More direct manipulations that alter the conditions of maternal tissues, such as water, nutrients or age, verify the role of maternal tissues in differential mating [e.g. (Marshall and Ellstrand 1988; Marshall and Fuller 1994; Lau and Stephenson 1994; Marshall and Diggle 2001; Haileselassie et al. 2005; Marshall et al. 2007)]. By and large, these studies have been performed on populations of routinely outcrossing plants. Little work has been done to explore variation in reproductive success in single and mixed pollinations in a historically selfing species.

In this study, we address two questions: (1) Is differential reproductive success based on the parental genetic distance in predominately selfing plants? (2) In the absence of early inbreeding depression, is there substantial maternal and/or paternal variation in reproductive success in natural populations? To do so, we performed diallel crosses in the predominately selfing plant, *Arabidopsis thaliana* (Arabidopsis). We chose seven accessions of Arabidopsis that cover a wide range of parental genetic distances as defined by neutral marker differences. These accessions were used for single pollinations in a full diallel crossing design, and seed yield was quantified. These same accessions were used for mixed pollinations in a modified diallel crossing design, where pollen from each accession was competed against a standard pollen competitor and proportion of seeds sired was quantified. Our results show that, in a system that has largely eliminated factors such as heterozygosity and inbreeding depression, there persists significant paternal and maternal variation in reproductive success. In the case of paternal variation in mixed pollinations, we argue that pollen performance is the most likely arbiter of differential reproductive success.

## Materials and methods

### Study system and plant growth

*Arabidopsis thaliana* (L.) Heynh. (Brassicaceae, formally Cruciferae) is an annual weed adapted to a wide range of habitats. Arabidopsis is native to Eurasia and North Africa but has become widely distributed throughout the Northern Hemisphere (Al-Shehbaz and O’ Kane 2002; Hoffmann 2002). Unlike close relatives, Arabidopsis is a predominately self-fertilizing species and displays the characteristic floral morphology: small flowers, lack of strong scent and close juxtaposition of anthers to stigma. Selfing rates in natural populations have been estimated to be greater than 97 % (Abbott and Gomes 1989; Bergelson et al. 1998; Bakker et al. 2006; Pico et al. 2008; Platt et al. 2010).

All plants were grown in identical, controlled environments. Briefly, plants were sown in 4.5” pots in Schultz premium potting soil with 10 plants per pot. Seeds were imbibed and cold-treated at 4 °C for 7 days in the dark to promote uniform germination. Plants were grown in Percival growth chambers (Percival Scientific, Inc., Perry Iowa) at 22 °C and subjected to 16-h 130-μE fluorescent lighting. Plants were watered every second day and fertilized twice every week.

### Arabidopsis accessions offer a range of defined genetic distances

For this study, we wanted to sample across a range of genetic distances to reveal patterns of success in reproduction. Many studies have used physical distance between populations as a loose correlate for genetic distance between plants [reviewed in (Waser 1993)], yet physical distance does not necessarily reflect genetic distance (Heywood 1991; Waser 1993; Hamrick and Godt 1996; Bell et al. 2010), and it is likely that genetic organization in space will vary in different populations and different species. This is especially the case for Arabidopsis, which shows complex patterns of natural variation at a variety of geographic scales (Sharbel et al. 2000; Nordborg et al. 2005; Stenoien et al. 2005; Beck et al. 2008; Pico et al. 2008; Bomblies et al. 2010; Platt et al. 2010; Horton et al. 2012). Arabidopsis populations, though predominately selfing, do not act as separately evolving asexual lineages, and no simple genome-wide phylogeny of accessions exists. Instead, only a small number of loci show monophyly for sets of accessions that form genetic clusters on a genome-wide scale (Nordborg et al. 2005). Within-region patterns are also complex. For example, Central Europe contains a “suture zone,” where genotypes from many different genetic clusters blend (Sharbel et al. 2000; Francois et al. 2008; Bomblies et al. 2010). Because of this complexity, instead of using geographical clusters of plants as a proxy for genetic distance, we chose to use direct measures of genetic variation that span a range from within-population to between-population values.

The genetic comparisons between the accessions was assessed as a measure of genetic dissimilarity of neutral markers, hereafter referred to as genetic distance (Willi and Van Buskirk, 2005; Bomblies et al. 2010). We compared DNA sequences from each accession from a large number of loci across the genome. We used the PCR-based Sanger sequencing results from 1214 manually curated sequenced fragment loci that are publicly available from the Nordborg Web site (https://cynin.gmi.oeaw.ac.at/home/resources/atpolydb/miscellaneous-data/alignments_042006.tar.z/view). This data set includes sequences from a total of 96 accessions, including the seven used in this study. See (Nordborg et al. 2005) for more information about an earlier version of this data set. We filtered the 1,214 locus alignments to exclude all positions that contained gaps or ambiguous bases in any of our seven accessions. Then, for each pair of accessions, we used the remaining 372,103 sites to quantify the percentage of sites that differed between pairs of sequences to create a genetic distance matrix (Table 1).

**Table 1:** Genetic distance of seven accessions of *Arabidopsis thaliana*

	Cvi-0 (%)	An-1 (%)	Kz-1 (%)	NFA-10 (%)	Ws-2 (%)	Bur-0 (%)	Col-0 (%)
Cvi-0	0	0.63	0.66	0.64	0.63	0.64	0.65
An-1	0.63	0	0.53	0.44	0.49	0.48	0.46
Kz-1	0.66	0.53	0	0.51	0.49	0.58	0.47
NFA-10	0.64	0.44	0.51	0	0.48	0.50	0.42
Ws-2	0.63	0.49	0.49	0.48	0	0.48	0.35
Bur-0	0.64	0.48	0.58	0.50	0.48	0	0.50
Col-0	0.65	0.46	0.47	0.42	0.35	0.50	0

Numbers listed are percent difference between accessions

We chose accessions that span a range of genetic differences from 0 (self-pollinations) to 0.66 % (Table 1). These values are comparable to values seen within and between geographically close populations of Arabidopsis (although each accession we chose is from a distinct population). For example, Bomblies et al. compared the genetic distance between 1,005 individuals within and between local populations of 77 closely spaced stands in Tubingen, Germany (Bomblies et al. 2010). Pairwise comparisons of genetic distance using single nucleotide polymorphism (SNPs), similar to our procedure described above, show that for between-population comparisons, there was a mean of 0.58 ± 0.09 % difference in the genotyped SNPs. The mean difference within populations, excluding identical individuals, was 0.35 ± 0.2 %.

For this study, the two accessions that were most similar were WS-2(CS22623) and Col-0(CS22625) that have a genetic distance of 0.35 %. The two accessions that were the most different were Cvi-0 (CS22614) and Kz-1(CS22606) that have a genetic distance of 0.66 %. The rest of the accessions were of intermediate genetic distance, Bur-0(CS22656), NFA-10 (CS22599) and An-1(CS22626) (Table 1). The standard pollen competitor accession, Col-NPTII, is an derivative of the Col-0 accession containing an integrated, intergenic kanamycin resistance marker. Col-NPTII is a F2 homozygous T-DNA insertion mutant obtained from the SIGnAL project (GenBank BZ377762) (Alonso et al. 2003). The intergenic T-DNA lies between the genes At1g28440 and At1g28450. The presence of the kanamycin resistance marker does not change the competitive performance of the pollen grain (Carlson et al. 2009). Since the Col accession is the parent strain for our standard pollen competitor, Col-NPTII, and presumably only differs based on the presence of the T-DNA, we treated Col-NPTII as Col for measures of genetic distance. We obtained all strains from the Arabidopsis Biological Resource Center (http://abrc.osu.edu/).

### Experimental pollinations

The methods for both types of pollinations were previously described and were performed on primary inflorescences of newly bolting maternal plants (Carlson et al. 2009). Briefly, we emasculate buds during stage 11–12 during development (Smyth et al. 1990). We allow pistils to mature to stage 14 before we perform mating assays. We harvest anthers from stage 14 flowers and visually inspect them for levels of dehiscence. We choose two anthers from each potential father and ready them on forceps. We use a stereomicroscope (Leica ZOOM2000) to better visualize the stigma when we apply pollen from the standard pollen competitor (Col-NPTII) on half the available surface area of the stigma. We then apply pollen from the competing accession on the remaining surface area. It has been noted in other systems that different methods of pollen application in mixed pollinations can lead to different results (Marshall and Folsom 1992; Mitchell and Marshall 1995). We use this method for consistency in pollen delivery. We complete each assay within 1 min. In previous work, we demonstrate consistent delivery of equal amounts of pollen in control mixed pollinations by competing Col-NPTII pollen and wild-type Col pollen on Col pistils, resulting in statistically equal amounts of progeny (Carlson et al. 2009). The data set for this experiment includes replications of these control mixed pollinations: seed set from mixed pollinations with pollen from Col-NPTII and Col-0 accessions on Col-0 pistils do not differ substantially from the expected 1:1 ratio: *χ* ^2^(1, *N* = 388) = 1.48, *p* = 0.223 (Table 3). Our single pollinations are performed as described above, except that all pollinating anthers are taken from a single accession.

For single pollinations, pollen from each of the seven chosen accessions was used to pollinate pistils from each accession in a 7 × 8 diallel mating design (we included pollen from the standard pollen competitor from the mixed pollination experiment). Mature siliques were then collected, individually split open, and seed yield was then counted (Table 2). We analyzed 494 pollinations, with each cell in the 7 × 8 design averaging 8.8 ± 1.8 successful pollinations. Mean seed yield ranged from 16.6 to 62.7 seeds.

**Table 2:** Seed yield results from single pollinations

Maternal accession	Paternal accession							
	Cvi-0	An-1	Kz-1	NFA-10	Ws-2	Bur-0	Col-0	Col-NPTII
Cvi-0	35.6 ± 3.4	20.6 ± 3.6	25.1 ± 4.9	29.3 ± 5.6	36.5 ± 1.8	37.2 ± 2.6	26.6 ± 4.8	23.6 ± 5.8
An-1	34.1 ± 4.2	31.4 ± 3.1	39.4 ± 2.8	30.6 ± 3.1	41.7 ± 3.9	***46.1*** ± ***2.1***	***49.7*** ± ***1.6***	36.7 ± 2.9
Kz-1	29.3 ± 4.4	31.8 ± 3.3	22.2 ± 3.0	32.3 ± 4.5	32.8 ± 3.4	28.5 ± 2.5	***38.0*** *±* ***4.9***	35.4 ± 3.5
NFA-10	44.2 ± 6.9	47.9 ± 4.3	62.7 ± 1.7	54.1 ± 4.1	50.3 ± 4.4	54.9 ± 5.4	42.5 ± 4.7	**34.7** ± **5.3**
Ws-2	27.3 ± 3.9	30.4 ± 4.2	27.1 ± 2.0	47.1 ± 4.6	34.4 ± 4.4	37.9 ± 5.4	20.9 ± 4.2	32.3 ± 4.1
Bur-0	28.1 ± 3.9	**16.6** ± **3.1**	34.0 ± 6.8	24.8 ± 3.0	21.4 ± 2.7	35.9 ± 6.0	26.2 ± 3.7	29.5 ± 4.0
Col-0	31.0 ± 4.9	30.1 ± 4.2	30.7 ± 4.3	34.7 ± 5.8	34.1 ± 4.1	38.0 ± 2.1	47.3 ± 5.0	34.0 ± 2.7

The mean seed yield and standard error of seeds sired in single pollinations. We compared seed yield from self-pollinations with outcross pollinations across maternal accessions via least significant difference pairwise comparisons. Bold = self-pollen shows greater seed yield than indicated outcross accession (*p* ≤ 0.05). Bolditalic = self-pollen shows less seed yield than indicated outcross accession (*p* ≤ 0.05)

For mixed pollinations, pollen from each of the seven accessions was placed in competition with pollen from the standard pollen competitor accession (Col-NPTII) on pistils from each of the seven accessions in a modified 7 × 7 diallel mating design. After mixed pollinations, mature siliques were collected and the seed paternity was assayed by growing on standard Murashige and Skoog media with sucrose containing 50ug/ml kanamycin (Murashige and Skoog 1962). Seeds that contain the NPTII gene grow normally. Those lacking the NPTII gene germinate, but the cotyledons turn nearly white and fail to develop true leaves. Proportion of seeds sired by paternal accessions from mixed pollinations were calculated as follows:

Proportion of seeds sired by paternal accession = # progeny sired by paternal accession/(# progeny sired by paternal accession + # progeny sired by standard competitor pollen).

We analyzed 433 mixed pollinations for proportion of seeds sired. Each cell in the 7 × 7 design averaged 8.8 ± 2.2 successful pollinations (Table 3). Mean proportion of seeds sired of the accessions ranged from 0.01 to 0.59.

**Table 3:** Proportions of seed sired from mixed pollinations

Maternal accession	Paternal accession						
	Cvi-0	An-1	Kz-1	NFA-10	Ws-2	Bur-0	Col-0
Cvi-0	0.02 ± 0.01	***0.12*** ± ***0.03***	***0.31*** ± ***0.08***	***0.24*** ± ***0.05***	***0.39*** ± ***0.04***	***0.44*** ± ***0.08***	***0.37*** ± ***0.07***
An-1	**0.08** ± **0.05**	0.34 ± 0.05	0.38 ± 0.06	***0.45*** ± ***0.05***	0.33 ± 0.05	0.34 ± 0.06	0.29 ± 0.03
Kz-1	0.39 ± 0.13	0.26 ± 0.04	0.35 ± 0.03	**0.22** ± **0.04**	0.38 ± 0.10	***0.47*** ± ***0.10***	***0.46*** ± ***0.06***
NFA-10	**0.06** ± **0.02**	**0.37** ± **0.05**	0.53 ± 0.07	0.41 ± 0.06	**0.33** ± **0.05**	**0.35** ± **0.06**	0.53 ± 0.04
Ws-2	**0.06** ± **0.03**	0.34 ± 0.03	0.29 ± 0.04	0.24 ± 0.03	0.29 ± 0.04	0.15 ± 0.05	***0.47*** ± ***0.08***
Bur-0	**0.01** ± **0.01**	**0.14** ± **0.04**	**0.11** ± **0.05**	0.32 ± 0.07	**0.19** ± **0.03**	0.32 ± 0.08	***0.40*** ± ***0.07***
Col-0	**0.12** ± **0.02**	**0.29** ± **0.07**	0.59 ± 0.04	**0.36** ± **0.04**	**0.28** ± **0.04**	**0.41** ± **0.04**	0.52 ± 0.06

The average proportion and standard error of seeds sired by the pollen from the indicated accession in mixed pollinations with a standard competitor pollen. We compared proportion of seeds sired from self-pollinations with outcross pollinations across maternal accessions via least significant difference pairwise comparisons. Bold = self-pollen shows greater proportion than indicated outcross accession (*p* ≤ 0.05). Bolditalic = indicated outcross accession shows greater proportion than self (*p* ≤ 0.05)

Some pollinations yielded no seeds. Although it is possible this is due to biological reasons, such as regulated fruit abortion, we often see this as a consequence of experimental manipulation. For example, in our experience, some pollinations fail due to damage to the flower sustained during emasculations. Because we cannot distinguish between these possibilities, and our main focus for this study is not regulated fruit abortion (a process we have not seen in Arabidopsis), we excluded any pollination that yielded no viable seed from our analysis.

### Statistical analysis

Diallel analysis produces six kin classes; full siblings, half siblings, reciprocal full and half siblings and the selfs, and allows the partitioning of variance in a data set into six components (Falconer 1989). We used the “bio” model of Cockerham and Weir (Cockerham and Weir 1977), with minor modifications, to partition the total phenotypic variance (*V*_phenotypic_) of seed yield and proportion of seeds sired into multiple components. We used the observational variance to causal variance transformation described by Montalvo and Shaw (Montalvo and Shaw 1994), and the terminology suggested by (Lyons 1996).

$$ V_{\text{phenotypic}} = \, V_{\text{maternal}} + \, V_{\text{paternal}} + \, V_{\text{joint}} + \, V_{\text{combined}} + \, V_{\text{interaction}} + \, V_{\text{environmental}} + \, \mu \, + \, G \, + \, D $$

*V*_maternal_ may involve variance originating with maternal environmental effects, cytoplasmic effects and/or maternal-specific nuclear effects. The similar is true for *V*_paternal_. *V*_joint_ represents the variance attributable to the parents, irrespective of whether it originates from the maternal or paternal side. This component was termed “Nuclear” by Cockerham and Weir (Cockerham and Weir 1977). *V*_combined_ represents the variance that is specific to particular parental combinations and has sometimes been termed “Dominance.” *V*_interaction_ includes the variance seen in parental combinations when the sex roles of the parents are reversed. *V*_environmental_ refers to residuals seen within cells in the design. *μ* is the population mean, a fixed effect. *G* is the genetic distance between paternal and maternal lines, treated as a fixed effect. Finally, *D* is the dummy variable, which indicates self-pollinations, also treated as a fixed effect. The last two variables allow us to assess the role parental genetic distance and self-pollination has on the variation in the data set. We chose to include self-crosses in our analyses, something that is often not done in diallels of outbreeding species because for species that experience inbreeding depression, self-cross results do not accurately reflect parental performance, and their inclusion can unduly influence variance estimates.

As has been noted by other researchers, the application of the “bio” model of Cockerham and Weir (Cockerham and Weir 1977) to postpollination traits such as seed yield is more limited than for traits that are analyzed in the F1 progeny of the Diallel cross (Cockerham and Weir 1977; Waser et al. 1995; Lipow and Wyatt 1999). Postpollination traits can include variance that may reside in the function of the haploid male and female gametophytes, as well as the triploid endosperm. Thus, the estimates of the “bio” model reflect combinations of interactions between parent, gametophytes and progeny, and must be interpreted with caution.

We were conflicted as to whether to treat the diallels as fixed- or random-effect models. On the one hand, since the accessions were not randomly chosen from a parent population, it may be proper to treat the analyses as a fixed-effect model (model I). In the end, we chose to treat the analyses as a random-effect model (model II) since the accessions were not systematically chosen based on phenotypes and may be seen as a random sampling of a species with widespread distribution.

Seed yield data from single pollinations were tested for normality using the Kolmogorov–Smirnov test *D* = 0.037, *p* > 0.05. Proportion of seeds sired data, however, fail tests for normality, despite Box–Cox transformation. Components were partitioned using restricted maximum likelihood (REML) approach in the PROC MIXED procedure in SAS. Variance components were constrained to nonnegative values, and the significance was estimated for each component using a likelihood ratio test.

Because parental shares in mixed pollinations did not conform to a normal distribution, interpretation of the components of variance, as well as the role of parental genetic distance in the diallel is problematic. To examine parental genetic distance using a nonparametric statistic, we performed a Spearman’s coefficient of rank correlation between parental genetic distance and proportion of seeds sired (after undergoing an arcsine transformation, to better normalize them) in SAS.

Because we were interested in trends among individual lines for seed yield in single pollinations, we performed a two-way ANOVA using GLM with maternal and paternal identity treated as fixed effects. We could then perform a least significant difference pairwise comparison to examine whether self-pollen differs from outcross pollen within each maternal accession. For similar reasons, we performed a test of equal proportions using GLM in R on data from mixed pollinations, with maternal and paternal identity treated as fixed effects. To investigate the differences between self- and outcross pollen, we performed a least significant difference pairwise comparison within each maternal accession. We applied an arcsine square root transformation on the proportional data to better normalize it. We modified all significance levels with a Bonferroni adjustment to account for multiple comparisons.

## Results

### Single pollinations reveal importance of maternal identity and lack of early inbreeding depression

We performed diallel analysis to partition the variance in single pollinations into separate components. Variation of seed yield in single pollinations is attributable to *V*_maternal_ and *V*_environmental_, with no significant variance contributed by *V*_paternal_, *V*_joint_, *V*_combined_ or *V*_interaction_ (Table 4). *V*_maternal_ contributed 16.6 % of the total variance, with the majority of variance attributable to *V*_environmental_ (82.6 %).

**Table 4:** Variance components for seed yield from single pollinations and proportion of seeds sired from mixed pollinations calculated after crossing individuals of *Arabidopsis thaliana* in diallels

Traits	*V*_maternal_	*V*_paternal_	*V*_joint_	*V*_combined_	*V*_interaction_	*V*_environmental_
Seed yield	16.6 (30.94)*	0.8 (1.53)	0 (0)	0 (0)	0 (0)	82.6 (153.96)
Proportion of seeds sired	6.2 (0.006)	17.9 (0.018)*	0 (0)	0 (0)	0 (0)	75.9 (0.076)

Parentheses contain values of restricted maximum likelihood estimates of variance components. Negative estimates were constrained to zero. Significance levels for individual components (except *V*_environmental_)

* *p* < 0.05

A standard way of measuring early-acting inbreeding depression is to compare seed yield from self-pollen to outcross pollen, with lower yields from self-pollen often attributed to abortion of homozygous offspring during embryo development because of the presence of paired deleterious recessive alleles. To assess potential effects of early-acting inbreeding depression on seed yield, our diallel analysis included a variable for self-pollinations; self-pollinations do not explain substantial variance in the data (*p* = 0.67). Consistent with this, when we included a variable for parental genetic distance into the diallel analysis, we found that parental distance does not explain substantial variance in the data (*p* = 0.92). Self-pollen does not display lower seed yield. Indeed, across the data set, self-pollen sired slightly higher than average seeds per fruit than outcross pollen (37.9, SEM 1.8 vs. 33.7, SEM 1.8, respectively).

Our model does not, however, allow us to distinguish whether there is no significant effect of selfing because these factors do not matter for seed yield in our populations, or because different maternal lines show different trends. For example, some maternal lines may show decreased seed yield in self-pollinations compared to outcross, but the significance of this may be obscured by other lines that show no effect, or show an opposite effect. To investigate trends across maternal lines, we conducted a two-way ANOVA using GLM. We find that paternal identity *F*(7, 438) = 3.37, *p* = 0.002, maternal identity *F*(6, 438) = 22.72 *p* < 0.001, and an interaction between the two *F*(42, 438) = 2.37, *p* < 0.001, all influence seed yield. Importantly, when we performed a pairwise comparison analysis between self- and outcross pollen across maternal accessions, we found that pairwise comparisons were generally not significant (Table 2). We find that self-pollen does not perform consistently worse (or better) in single pollinations. In 3 maternal lineages (Col-0, Cvi-0 and Ws-2), we find no statistical difference between self- and outcross pollen. In 2 maternal lineages (Bur-0 and NFA-10), self-pollen performs significantly better than one of the seven outcross pollen. In 2 maternal lineages (Kz-1 and An-1), self-pollen performed significantly worse than one and two of the seven outcross pollens, respectively.

### Mixed pollinations reveal importance of paternal identity and lack of cryptic self-incompatibility

Diallel analysis of mixed pollinations revealed significant variation in proportion of seeds sired attributable to *V*_paternal_ and *V*_environmental_ (Table 4). *V*_paternal_ contributed 17.9 % of the total variance, with the majority of variance contributed by *V*_envrinomental_ (75.9 %). This indicates that paternal identity plays a significant role in reproductive success in competitive pollinations.

To assess the role of selfing and parental genetic distance on reproductive success, our diallel analysis included variables for self-pollen and parental genetic distance. Self-pollen has no significant effect on proportion of seeds sired in mixed pollinations (*p* = 0.30). Parental genetic distance also had no significant effect (*p* = 0.21). Because of the nonnormality of the mixed pollination data set, we also tested for correlations between parental genetic distance and proportion of seeds sired using a nonparametric correlation. We performed Spearman’s coefficient of rank correlation and determined that no relationship exists between parental genetic distance and proportion of seeds sired in mixed pollinations (*r* = −0.242, *n* = 49, *p* = 0.094).

In order to determine whether nonsignificance in our diallel analysis is due to different trends across different maternal lineages, we performed a test of equal proportions and found that paternal identity *F*(6, 384) = 21.46, *p* < 0.001, maternal identity *F*(6, 384) = 7.66 *p* < 0.001, and an interaction between the two *F*(36, 384) = 2.93, *p* < 0.001, all influence proportion of seeds sired in mixed pollinations. As with single pollinations, self-pollen averaged slightly better performance compared to outcross pollen (0.34 of progeny, SEM = 0.02 vs. 0.30 of progeny, SEM = 0.01, respectively). We performed a pairwise comparison analysis between self- and outcross pollen. The patterns we see are complex, and not consistent among maternal lineages (Table 3). In some maternal lineages, such as Col-0, Bur-0 and NFA-10, self-pollen performs statistically better than outcross pollen most of the time. In one lineage, Cvi-0, outcross pollen outperforms self-pollen in all cases. Normally, this trend would be suggestive of early-acting inbreeding depression, but it is worth noting that Cvi-0 pollen is a fairly poor competitor on all maternal accessions. In the remaining lineages, An-1, Kz-1 and Ws-2, neither self- nor outcross pollen performs statistically better the majority of the time.

## Discussion

### Differential reproductive success is not based on the parental genetic distance in predominately selfing plants

Parental genetic distance, the genetic similarity between a pollen donor and the receiving maternal plant, has been successfully used as a metric to predict differential reproductive success under several different mating schemes. These predictions are fairly straightforward for plants that exhibit physiological self-incompatibility, where self-pollen fails [reviewed in (Franklin-Tong 2008)], and in mixed pollinations of conspecific and heterospecific pollen, conspecific pollen has advantages (Howard 1999; Williams et al. 1999; Yost and Kay 2009; Montgomery et al. 2010; Baldwin and Husband 2011).

For pollinations of conspecific self-compatible plants, there are also clear trends. Although reports vary somewhat, generally self-pollinations result in relatively poor reproductive success in predominately outcrossing plants, likely as a result of inbreeding depression. With increases in selfing, autogamous plants go through a purging of genetic load that would presumably reduce early embryonic lethality and the selective pressures of inbreeding that would favor outcross pollen. It seems reasonable to predict that this would relieve bias against self-pollen. Our data support these conclusions. We find that self-pollen is not at a consistent disadvantage over outcross pollen and often performs better. And this conclusion is consistent with studies on other autogamous plants. Weller et al. explored early life stage measures of inbreeding depression in the predominately selfing plant *Schiedea viscosa*. Seed yield from self-pollinations was not statistically distinguishable from outcross pollen in the four populations that were tested (Weller et al. 2005).

The predictions of reproductive success are less clear with increasingly unrelated plants. Relatively, few studies have addressed reproductive success from larger spatial scales (and thus, presumably parental divergence), and the results from these studies are complex. Reproduction measures often decline with pollen delivered from increasing distance from the maternal plant (Price and Waser 1979; Waser and Price 1991; Baker and Shore 1995), although there are a number of studies that show inconsistent or little difference in reproductive success from distant populations (Schlichting and Devlin 1992; Hauser and Loeschcke 1994; Hauser and Siegismund 2000; Jolivet and Bernasconi 2007). In cases when distantly related pollen donors result in weaker reproductive success, one possible explanation is outbreeding depression. Although very little work has been done investigating outbreeding depression in predominately selfing angiosperms, there are some predictions that the effects of outbreeding depression should be stronger. Autogamy is expected to increase genetic variation between lineages and restrict recombination. This in turn may exacerbate the development of co-adapted gene complexes between populations, the breaking of which can lead to reduced fitness in progeny (Templeton et al. 1986). There is empirical support for this idea. Progeny fathered by close siblings showed a clear fitness advantage over progeny fathered by unrelated males in the predominately selfing haplodiploid beetle, *Xylosandrus germanus* (Peer and Taborsky 2005). Similar results have been documented in ferns (*Asplenium ruta*-*muraria)* and fig wasps (*Platyscapa awekei*) (Schneller 1996; Greeff et al. 2009). In this study, we find no relationship between parental distance and reproductive success in single pollinations. This result aligns with similar work done in *L. crassa* (Lyons 1996). We also find no consistent relationship between parental distance and reproductive success in mixed pollinations.

Thus, we find that patterns of reproductive success in Arabidopsis are not consistent with influences from early-acting inbreeding or outbreeding depression. This allows us to ask the question whether, in the absence of these forces, there persists substantial maternal and paternal variation in reproductive success.

### Paternal variation in mixed pollinations is likely due to differential pollen germination and tube growth dynamics

Diallel studies of single pollen donor pollinations of natural populations uncover little significant variance attributable to a paternal effect, consistent with our single pollen donor experiment. Mixed pollination experiments offer a more subtle measure of postpollination reproductive success and have been more successful in revealing paternal effects. Our diallel analysis of proportion of seeds sired in mixed pollination shows 17.9 % of the phenotypic variance attributable to paternal effect. This supports the hypothesis of Waser et al. that paternal variance in reproductive success would be uncovered if diallel experimental designs include treatments with multiple pollen donors (Waser et al. 1995). It is worth noting that, although studies looking at seed yield using the “bio” model have not uncovered substantial paternal variance, models that include only male, female and interaction effects often do [e.g. (Marshall 1988, 1998; Mitchell and Marshall 1998; Schlichting and Devlin 1992)]. We also see paternal effects with our two-way ANOVA of seed yield in single pollinations. As has been noted by Waser et al., this may be a result of simpler models not providing formal partitioning of variance that would allow assessment of the extent of the contribution of the paternal identity to the overall amount of phenotypic variance, making the diallel a more conservative estimate of parental effect (Waser et al. 1995).

Although we do not directly assess pollen performance measures in this study, the differences we see in single and mixed pollinations are not due to differential pollen viability of the accessions we tested (accessions show little difference in viability, as measured by Alexander’s stain, data not shown). The differences we see are also unlikely due to early embryonic lethality as a result of inbreeding depression. A standard way this is assayed is by comparing seed yield from self- and outcross pollen, with lower yields from self-pollen attributed to failed embryo development. We find that self-pollen performs slightly better than outcross pollen in both single and mixed pollinations. Based on this, it seems reasonable to hypothesize that the paternal variation we see in mixed pollinations is due to differential pollen germination and tube growth dynamics (although there remains the possibility of early embryo lethality playing a role due to unknown mechanisms). Indeed, it has been hypothesized that pollen tube growth rates may be more varied between predominately selfing plant populations as a result of the lack of strong selective pressures that accompany pollen competition among more heterogeneous pollen cohorts (Mazer et al. 2010; Taylor and Williams 2012). Should this be the case, genetic studies of male-mediated differential reproductive success in Arabidopsis should likely focus attention on genes that affect these processes (Carlson et al. 2011).

### Maternal variation in single pollinations is consistent with earlier studies

We also show that phenotypic variance in seed yield can be attributed to maternal effect (16.6 %). This result is consistent with several other studies of natural populations. These studies contrast with our study in that they have been performed on individuals randomly chosen from geographically centralized populations, and have largely been performed on plants that are predominately outcrossing. For example, Waser et al. studied seed yield in populations of *Ipomopsis aggregata*, a predominately outcrossing plant with late-acting self-incompatibility, and showed that maternal variance in seed yield ranges between 12 and 35 % (Waser et al. 1995). Similarly, Good-Avila and Stephenson show that in the partially self-incompatible *C. rapunculoides*, maternal variation accounts for 36 % of the phenotypic variance in seed yield (Good-Avila et al. 2003). Lipow and Wyatt show that maternal effect accounts for between 20.5 and 37.1 % of the phenotypic variance in seed yield in the largely outcrossing *A. incarnate* (Lipow and Wyatt 1999). Finally, Lyons contrasted the phenotypic variances of populations of outcrossing *L. crassa* with populations that predominately self. Both populations show significant maternal effect on seed yield (Lyons 1996). What is clear is that for plants that outcross and predominately self, maternal identity both strongly influences and varies in seed yield in natural populations.

Our conclusions come with several important qualifiers. First, our estimates of genetic distance between accessions utilize a large number of genetic markers, but what actually matters is the divergence that has occurred in gene loci that affect the process of reproduction. Because we do not yet have an understanding of the genetic basis for natural variation in this process [although see (Carlson et al. 2011)], we are left with our general measures of genome divergence as a proxy. Second, postpollination reproductive success can occur at a variety of physiological stages that include differential pollen viability, pollen germination, pollen tube growth rate, fertilization and abortion [for review, see (Marshall and Folsom 1991)]. With the exception of assessing differential pollen viability, an unlikely component of the patterns we see (data not shown), we did not dissect these stages in this study. It is possible that these different stages experience different selective pressures. For example, it is possible that self-pollen germinates at higher levels on self-pistils, but self-pollen tubes grow slower through styles. The present study is not designed to distinguish these features. Finally, our focus in this experiment was on the role of parental genetic distance in reproductive success, independent of any role local adaptation to environmental conditions may play in differential reproductive success.

## Conclusion

The difficulty in studying variance in mating in outcrossing populations is a result of segregating heterozygosity and inbreeding depression. In historically selfing populations, evolution has largely eliminated these factors. This offers us the opportunity to explore naturally occurring variation in reproductive success. We show that population-level genetic similarities, including selfing, do not correlate with nonrandom mating, yet there is still significant paternal variance under competition. This suggests two things. Since these differences are unlikely due to early-acting embryo lethality or pollen viability, this implicates natural variation in pollen germination and tube growth dynamics. This also strongly suggests fixation of pollen performance genes in populations, offering a focus for future genetic studies in differential reproductive success.
